# The spatial distribution of potentially toxic elements in the mountain forest topsoils (the Silesian Beskids, southern Poland)

**DOI:** 10.1038/s41598-023-50817-7

**Published:** 2024-01-03

**Authors:** Oimahmad Rahmonov, Michał Sobala, Dorota Środek, Dominik Karkosz, Sławomir Pytel, Małgorzata Rahmonov

**Affiliations:** Institute of Earth Sciences, Faculty of Natural Sciences, University of Silesia in Katowice, Będzińska 60, 41-200 Sosnowiec, Poland

**Keywords:** Forest ecology, Ecosystem ecology, Geochemistry, Environmental impact, Element cycles

## Abstract

Progressive industrialisation and urbanisation in recent decades have dramatically affected the soil cover and led to significant changes in its properties, which inevitably affect the functioning of other components of the forest ecosystems. The total content of Pb, Cd, Zn, Fe, Cr, Cu, Ni, As, and Hg was studied in twenty-five plots at different heights in the topsoil (organic and humus horizons) formed from the Carpathian flysch in the area of the Silesian Beskids (Western Carpathians). The aim of this article is to analyse the spatial distribution of potentially toxic elements in the mountain forest topsoil in different types of plant communities and to determine the relationship between altitude and potentially toxic elements contamination. The soils studied are acidic or very acidic, with an average range of 3.8 (H_2_O) and 2.9 (KCl). Concentrations of the metals Cd, Zn, Fe, Cr, Cu, Ni, and Hg on the plots that were analysed are within the range of permissible standards for forest ecosystems in Poland, while Pb and As exceed the permissible standards for this type of ecosystem. Spearman’s rank correlation coefficient showed a high correlation between Fe–Cr (r(32) = 0.879, Pb-Hg r(32) = 0.772, Ni–Cr r(32) = 0.738, Zn-Cd r(32) = 0.734, and Cu-Hg r(32) = 0.743, and a moderate statistically significant positive correlation between Cu-Pb r(32) = 0.667 and As-Pb r(32) = 0.557. No correlation was found between altitude and the occurrence of potentially toxic elements. The geo-accumulation index (Igeo) index, on the other hand, indicates that Pb, As, and Cd have the highest impact on soil contamination in all study plots: it classifies soils from moderately to strongly polluted. The enrichment factor (EF) obtained for As and Hg indicates significant-to-very high enrichment in all areas studied. The potential ecological risk index (PLI) calculated for the sites indicates the existence of pollution in all areas examined. The highest risk categories (considerable to very high) are associated with cadmium and mercury.

## Introduction

Several factors influence the concentration and distribution of trace metals in soil, such as soil particle size distribution, organic matter content, drainage, soil horizon, and vegetation^1,2^. The overall potentially toxic elements content of the soil also depends on the geochemical character of the parent material, as the soil inherits a certain quantity of elements from its parent rock, which are then redistributed by pedological processes^3–6^. The natural background of potentially toxic elements in uncontaminated soils is usually at the level of trace amounts, following their low content in most of igneous and sedimentary rocks. Only mafic and ultramafic rocks are known to be a rich source of some potentially toxic elements, for example, nickel and chromium^1^.

Rapid industrialization and urbanization during recent decades have been affecting dramatically soil properties and have led to large discharges of pollutants, which inevitably affects the health of the soil, ecosystems and human population^7^. In the last few centuries, and particularly in the last few decades, human activity has continuously increased the level of potentially toxic elements circulating in the environment. Therefore, contamination of ecosystems with trace elements has become an important topic of investigation, since many trace metals are toxic to terrestrial and aquatic organisms. This concerns mainly urban areas that are characterized by an increased content of many metals, especially in the surface layer of ground^5,6,8,9^. There is a large spatial variation of metal content mainly due to the ways the urban area develops and functions^10–12^. Nevertheless, soil potentially toxic elements pollution has also become an urgent environmental concern in non-urban areas^13–15^. This problem concerns not only agricultural land^16,17^ but also managed forests^4,18^ and protected areas^19,20^. This is because the majority of potentially toxic elements are emitted into the air together with dust particles, on which they are adsorbed and then transported over distances of hundreds of kilometres^21^. Then they can be deposited into the soil environment and on the surface of plants along with precipitation^22^.

The main source of potentially toxic elements emissions released into the environment is anthropogenic emissions and emissions from natural sources related to geological processes. Potentially toxic elements affect vegetation: their impact can be toxic, but it should be emphasized that some of the potentially toxic elements, such as zinc, copper and nickel, perform important physiological functions in plants and are necessary for the proper conduct of metabolic processes. The degree of toxicity of potentially toxic elements is influenced by many factors, including: time of exposure, bioavailability, interactions with other metals in the soil, nutritional status of a given plant, its health status, type of plant, location, age and presence and type of mycorrhization^23,24^.

In recent years, Europe's coniferous forests, mainly spruce, have been hit by mass extinction. This has been recorded in Germany^25^, Slovenia^26^, and former Czechoslovakia^27^, as well as in Poland^28^. Forest complexes suffered extensive degradation in the Beskids, a mountain range in the Carpathian Mountains (Central Europe), in the last decades of the twentieth century. The reasons for this situation are complex and some authors^18,29^ associate the extinction of the forests in the Carpathians mainly with rapid industrialization after World War II and the neglect of environmental protection during the socialist period, which resulted in increased pollution^28,30,31^. To a great extent, acid gas pollution and metal-bearing dust contributed to the poor condition of Poland’s forests. Acidic gaseous pollutants and dust, which include potentially toxic elements, cause many adverse changes in living organisms. Metal ions, especially Cd and Pb, are potent inhibitors of enzymes, affect the uptake of essential micro and macronutrients, and disrupt photosynthesis and other metabolic processes^32,33^.

According to some authors, the presence of potentially toxic elements in the soil is one of the reasons for the extinction of spruce stands throughout the Carpathians^34,35^. Other authors, on the other hand, argue that it cannot be conclusively established that potentially toxic elements play a crucial role in the extinction of spruce stands in the Polish Carpathians^18,36^. The authors of this study hypothesised that the level of contamination with potential toxic metals of soils under forest ecosystems may have influenced the degradation of spruce stands. Therefore, the research aimed to analyze the spatial distribution of potentially toxic elements in the mountain forest topsoil along the valley transverse transect in the Silesian Beskids (southern Poland). The research involves three main stages, namely: (1) analysis of the influence of environmental factors on the morphology and basic properties of topsoils, (2) analysis of the spatial variability of potentially toxic elements, and (3) assessment of the environmental risk based on selected indicators.

## Materials and methods

### Study area

**Figure 1:**
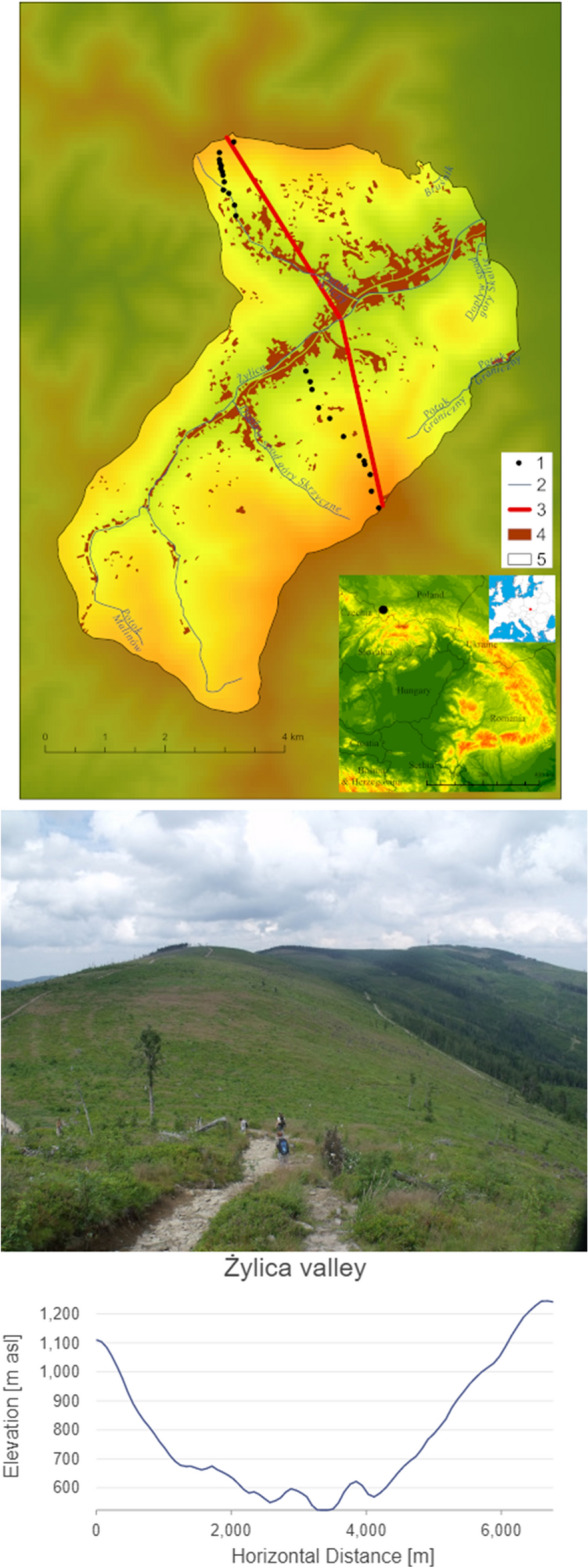
Study area and the transverse profile of Żylica valley. Explanations: 1, study sites; 2, watercourse; 3, transverse profile; 4, buildings; 5, the town of Szczyrk. Source: own elaboration based on Shuttle Radar Topography Mission (SRTM) https://earthexplorer.usgs.gov/ using ArcGIS Pro. Photo by Michał Sobala.

The study area is located in the vicinity of intensively developed industrial centres. The impact of air pollution from Upper Silesia, northern Moravia, Kraków and Bielsko-Biała is significant^37^. The detailed study was conducted in Żylica valley in the Western Carpathians (Poland) (Fig. 1). The town of Szczyrk, an important tourist centre in the region, is located in this valley. The basement of this area is made from formations of the Carpathian Flysch Belt that consists of alternating deposits of claystones, shales and carbonate-free sandstones. The area spans over three vertical climatic zones, namely, moderate warm (with mean temperature > 6 °C), moderate cool (4–6 °C) and cool (< 4 °C). Precipitation on the highest ridges (Skrzyczne) reaches 1300 mm per year^29^.

This area has been inhabited since the turn of the fifteenth and sixteenth centuries. At that time, Vlach shepherds came to this area, founding new settlements at higher elevations and forming glades by slashing and burning the forest for sheep to graze^38^. For this reason, humans have exerted a strong influence on land use and land cover. From the end of the seventeenth century until the mid-nineteenth century, the expansion of buildings, meadows, pasturelands and arable fields occurred^39^. In the mid-nineteenth century, mountain grazing started to collapse as a result of industrial development and the intensification of forest management connected with the Industrial Revolution. The abolition of serfdom and the stagnation in the sale of sheep products also had an influence. All these factors stimulated the afforestation of some mountain pastures by the second half of the nineteenth century. As a result, the surface of mountain pastures and glades decreased. This process has continued up to the present day^40,41^. The development of Szczyrk as a holiday and tourist site began in the inter-war period. Currently, Szczyrk is one of the most important winter holiday resorts in Poland and offers all-year-round resting and recreation conditions^42^. Consequently, one of the city's problems is excessive car traffic and low emissions, which are the sources of air pollutions^43^.

### Soil sampling

**Figure 2:**
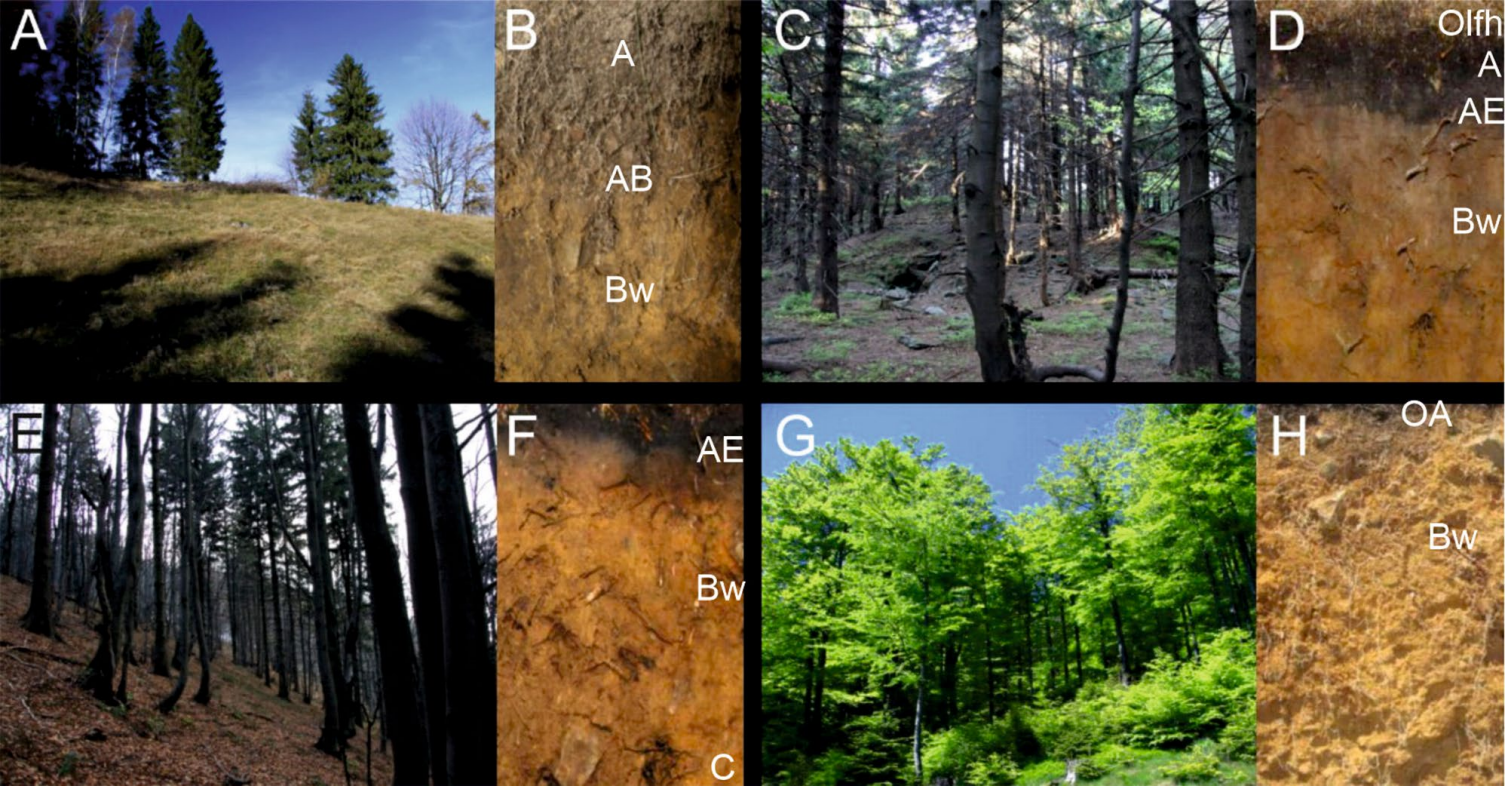
Dominant plant community types and the developed soil profiles under them: (**A**, **B**)—*Nardetum strictae*, (**C**, **D**)—*Plagiothecio-Piceetum*, (**E**, **F**)—*Abieti-Picetum (montanum)*, (**G**, **H**)—*Dentario glandulosae-Fagetum*. Photos by Oimahmad Rahmonov.

Soil samples were taken at intervals averaging 50 m from the peaks towards the valley at different altitudes (Fig. 1). Field study was conducted in October 2021. Three soil samples were taken from each site (96 samples were taken), and the findings include the average of the values from these samples. Soil samples from the organic horizons (O, including its sub horizons Ol/fh) and humus horizon (A) from 25 sites representing plant communities were submitted for laboratory analysis (Fig. 2). Twelve samples were taken from the organic horizon with sub horizons (O, Olfh) and 20 from the humus horizon (A). Not all sites contained organic horizons, and their absence was due to the presence of steep slopes subject to erosion. The thickness of the organic and humus horizons varied (Table 1). In the tables, samples numbered 1 to 12 represent the direction Szrzyczne peak (1252 m a.s.l.)—Szczyrk (640 m a.s.l.), while samples numbered 13–25 cover the direction Klimczok peak (1050 m a.s.l–Szczyrk (600 m a.s.l.) (Fig. 1).

**Table 1:** Organic carbon content and soil reaction (pH) under particular plant communities at the study sites.

Sections	Plot no.	Soil horizon	Thickness of horizon [cm]	Altitude m.a.s.l.	Plant communities	pH- H_2_O	pH- KCl	C_org_ [%]	Loss of ignition [%]
Skrzyczne–Szczyrk	1	A	20	1252	*Nardetum strictae*	3.3	2.6	9.2	21.1
	2	Olfh	5	1202	*Plagiothecio-Piceetum*	3.9	2.7	30.9	59.5
	3	A	5	1095	*Plagiothecio-Piceetum*	3.3	2.6	14.4	29.3
	4	A	5	1040	*Luzulo luzuloidis-Fagetum*	3.2	2.5	14.6	28
	5	A	4-0	1002	*Luzulo luzuloidis-Fagetum*	3.6	2.8	17.9	31.1
	6	A	10	958	*Luzulo luzuloidis-Fagetum*	3.6	2.8	17.4	28.1
	7	Olfh	4-0	870	*Abieti-Picetum (montanum)*	4.2	3.6	41.9	72.1
		A	10	870	*Abieti-Picetum (montanum)*	3.8	3.0	10.2	23.3
	8	A	5	880	*Abieti-Picetum (montanum)*	3.6	2.9	12.5	27.6
	9	A	1-0	780	*Luzulo luzuloidis-Fagetum*	3.6	2.8	20.3	41.9
	10	A	6	720	*Prunello-Plantaginetum*	4.1	3.5	2.6	5.9
	11	Ol/Ofh	8	680	*Abieti-Picetum (montanum)*	4.1	3.4	43.1	89.01
		A	23	680	*Abieti-Picetum (montanum)*	3.6	2.7	14.1	26.3
	12	A	20	640	*Arrhenatheretum elatioris*	4.9	3.9	3.0	8.9
Klimczok–Szczyrk	13	Olfh/A	6	1050	*Plagiothecio-Piceetum*	3.2	2.5	16.6	36.1
	14	A	13	1020	*Luzulo luzuloidis-Fagetum*	3.7	2.9	7.0	12.6
	15	Ol/Ofh	7	1000	*Abieti-Picetum (montanum)*	3.7	2.9	24.9	70.9
		A	14	1000	*Abieti-Picetum (montanum)*	3.6	2.7	5.1	9.9
	16	Olfh	2	990	*Dentario glandulosae-Fagetum*	3.6	2.9	12.8	24.2
	17	Olfh/A	7	980	*Dentario glandulosae-Fagetum*	4.4	3.5	37.1	83.2
		A	11	980	*Dentario glandulosae-Fagetum*	3.6	2.8	10.3	17
	18	Ol	2-0	940	*Abieti-Picetum (montanum)*	4.2	3.3	27.7	66.8
		A	9	940	*Abieti-Picetum (montanum)*	3.6	2.7	18.6	29.2
	19	A	4	920	*Abieti-Picetum (montanum)*	3.5	2.7	20.1	39.9
	20	Ol/Ofh	3,5	900	*Luzulo luzuloidis-Fagetum*	3.7	3.0	37.3	85.8
		A	15	900	*Luzulo luzuloidis-Fagetum*	3.4	2.7	24.9	52.1
	21	Ofh	4-0	880	*Abieti-Picetum (montanum)*	3.3	2.6	21.1	41.8
	22	Ol/Ofh	9	860	*Dentario glandulosae-Fagetum*	3.5	2.9	16.0	26.4
	23	Ol/Of	5	770	*Dentario glandulosae-Fagetum*	3.6	2.8	26.2	72
		A	5	770	*Dentario glandulosae-Fagetum*	3.8	3.0	9.3	14.2
	24	A	3-0	680	*Dentario glandulosae-Fagetum*	4.2	3.4	6.6	14.9
	25	A	5	600	*Dentario glandulosae-Fagetum*	3.9	2.8	34.2	64.2

### Laboratory analysis

The pH values were measured potentiometrically in H_2_O and in 1N KCl using a glass electrode. The following measurements and methods were carried out: total organic carbon (Corg.) according to Tiurin’s method and loss of ignition^44^.

The total composition of potentially toxic elements Pb, Cd, Zn, Fe, Cr, Cu, Ni, As, and Hg in soil were measured using ICP-OES (inductively coupled plasma op-tical emission spectrometry) after wet mineralization in nitric and hydrochloric acid (3HCl + HNO_3_). The analyses were performed in the ACME Laboratory (Vancouver, Canada) using AQ250_EXT procedures and 5 g samples. All soil samples were analysed in triplicate for all the parameters investigated, and mean values were calculated.

#### Estimating pollutant impact

To determine the potentially toxic elements concentration, their enrichment in soil, and potential contamination level, we calculate chemical indexes used in soil contamination studies^45–47^: geo-accumulation index (Igeo), enrichment factor (EF), contamination factor (CF), pollution load index (PLI), and potential ecological risk index (RI). These indices made it possible to show the state of soil enrichment and contamination from different elevations.

#### Index of geo-accumulation

The geo-accumulation index (Igeo) allows for assessing the degree of metal contamination or pollution in soil. It was calculated using a formula given by Okedeyi et al.^46^:

1 $$Igeo = log_{2} \left( {\frac{{C_{n} }}{{1.5B_{n} }}} \right),$$

where C_n_ is the content of the element in the studied sample and B_n_ is the concentration of the same element in the upper continental crust^48^. The chemical composition of the upper continental crust is a standard reference point used in calculating chemical indexes applied in various kinds of environmental studies^49–53^. We used the 1.5 factor to minimize the effect of possible variations in the background values^54^. The seven contamination classes can be assigned based on the increasing value of the geo-accumulation index^55^:

- Igeo < 0—uncontaminated
- 0 ≤ Igeo < 1—uncontaminated to moderately contaminated
- 1 ≤ Igeo < 2—moderately contaminated
- 2 ≤ Igeo < 3—moderately to highly contaminated
- 3 ≤ Igeo < 4—highly contaminated
- 4 ≤ Igeo < 5—highly to very highly contaminated
- 5 ≤ Igeo—very highly contaminated.

#### Enrichment factor

To estimate the degree of metal enrichment, we calculate the enrichment factor (EF). This method normalizes the content of metals with respect to a sample reference metal^55^. In this work, we used Fe as a reference metal for all calculated factors for potentially toxic elements, a recommended procedure that was used previously in similar studies^46,56–59^. The EF was defined using the formula:

2 $$EF = \frac{{\left[ {{\raise0.7ex\hbox{${C_{metal} }$} \!\mathord{\left/ {\vphantom {{C_{metal} } {C_{normalizer} }}}\right.\kern-0pt} \!\lower0.7ex\hbox{${C_{normalizer} }$}}} \right]soil}}{{\left[ {{\raise0.7ex\hbox{${C_{metal} }$} \!\mathord{\left/ {\vphantom {{C_{metal} } {C_{normalizer} }}}\right.\kern-0pt} \!\lower0.7ex\hbox{${C_{normalizer} }$}}} \right]control}},$$

where C_metal_ is the content of metal being examined and C_normalizer_ is the selected normalizer concentration in soil and the control sample^48^. The five categories can be distinguished based on the enrichment factor^60^:

- EF < 2—deficiency to minimal enrichment
- 2 ≤ EF < 5—moderate enrichment
- 5 ≤ EF < 20—significant enrichment
- 20 ≤ EF < 40—very high enrichment
- 40 ≤ EF—extremely high enrichment

#### Contamination factor

The contamination factor (CF) was calculated using the equation:

3 $$CF = \frac{{C_{n} }}{{B_{n} }},$$

where C_n_ is the element content in the soil, and B_n_ is the same element concentration in the average composition of the continental crust^48,61^. The background values amount to: 30.9 g kg^−1^ (Fe), 527 mg kg^−1^ (Mn), 35.0 mg kg^−1^ (Cr), 52.0 mg kg^−1^ (Zn), 18.6 mg kg^−1^ (Ni), 14.3 mg kg^−1^ (Cu), 17.0 mg kg^−1^ (Pb), 2.00 mg kg^−1^ (As), 0.10 mg kg^−1^ (Cd), and 0.06 mg kg^−1^ (Hg)^48^. It shows a degree of contamination related to the crustal composition. It can be distributed into four classes depending on the value:

- CF < 1—low contamination factor
- 1 ≤ CF < 3—moderate contamination factor
- 3 ≤ CF < 6—considerable contamination factor
- 6 ≤ CF—very high contamination factor

#### Pollution load index

The pollution load index (PLI) was estimated based on the contamination factors according to the formula:

4 $$PLI = \sqrt[n]{{CF_{1} \times CF_{2} \times CF_{3} \times \cdots \times CF_{n} }},$$

where n is the number of the contamination factors^47^. To calculate PLI, we used the five highest contamination factors suggested by Tomlinson et al.^62^. This index shows the potentially toxic elements contamination, and it can assume values < 1 (absence of pollution) or 1 < (existence of pollution)^62^.

#### Potential ecological risk index

The potential ecological risk index (RI) allows the evaluation of the potentially toxic elements impact on the environment. It was calculated using the formula:

5 $$RI = \sum E_{r}^{i} ,{ }$$

where $E_{r}^{i}$ is the potential ecological risk factor of the specific element^47^. The $E_{r}^{i}$ was calculated using the equation:

6 $$E_{r}^{i} = T_{r}^{i} \times CF,$$

where ${T}_{r}^{i}$ is the toxic response factor of the metal given by Zhu et al.^63^. The ecological risk can be divided into five classes^64^:

-  < 40—low
- 40 ≤< 80—moderate
- 80 ≤< 160—considerable
- 160 ≤ < 320—high
- 320 ≤ —very high.

and the risk index can be classified as^64^:

- RI < 150—low risk
- 150 ≤ RI < 300—moderate risk
- 300 ≤ RI < 600—considerable risk
- 600 ≤ RI—very high risk.

### Statistical analyses

In order to analyse similarities in the potentially toxic elements content of soil under different plant communities, cluster analysis was employed, using the method described by Ward^65^. This method allows multiple objects to be combined without a prior indication of the number of clusters, under the initial assumption that each object is a separate cluster, and then the objects most similar to each other are grouped together until a single cluster containing all observations is formed. The end result is a dendrogram, i.e. a tree hierarchy of the set, which illustrates, among other things, the overall similarity structure^66^. Thus, cluster analysis is a data analysis tool, the aim of which is to arrange objects into groups in such a way that the degree of association of objects with objects belonging to the same group is as high as possible and with objects from other groups is as low as possible.

The distance of the new cluster from each remaining cluster is determined by the formula:

7 $$D_{pr} = {\text{ a}}_{{1}} *{\text{ d}}_{{{\text{pr}}}} + {\text{ a}}_{{2}} *{\text{ d}}_{{{\text{qr}}}} + {\text{ b }}*{\text{ d}}_{{{\text{pq}}}}$$

r—takes the cluster numbers different from “p” and “q”.

D_pr_—distance of the new cluster from the cluster numbered “r”.

d_pr_—distance of the original cluster “p” from the cluster “r”.

d_qr_—distance of original cluster “q” from cluster “r”.

d_pq_—mutual distance of primary clusters “p” and “q”.

a1, a2, b—parameters, which have formulas in Ward’s method:

8 $$a_{1} = \frac{{n_{p} + n_{r} }}{{n_{p} + n_{q} + n_{r} }} a_{2} = \frac{{n_{q} + n_{r} }}{{n_{p} + n_{q} + n_{r} }} b = \frac{{ - n_{r} }}{{n_{p} + n_{q} + n_{r} }}$$

In these formulas, ‘n’ denotes the abundance of individual objects in each site.

The Spearman’s rank correlation coefficient is used to analyse the interdependence of objects in terms of a two-dimensional trait (X, Y). Assuming that we are examining n objects described by two characteristics, these objects must be ordered with regard to the values of each characteristic separately^66^. Spearman’s rank correlation coefficient was applied to check whether there was any relationship between the concentrations of the elements analysed and altitude. This is the coefficient used on samples that do not meet the assumptions of normality. The statistical significance of the Spearman correlation coefficient data was determined using the Spearman rank correlation test. The exact values of the correlation coefficient were calculated for alpha = 0.001, 0.01 and 0.05. All statistical analyses were performed using SPSS Statistics software**.**

## Results

### Soil properties

According to the international classification, analysed soils belong to the Inceptisol (USDA soil taxonomy, 2014)^67^ and Cambisol (World Reference Base for Soil Resources WRB 2015)^68^. According to the Systematic of Soils of Poland^69^, the soils analysed were defined as acidic brown soils (Dystric Cambisols), which occur both under forest communities (*Plagiothecio-Piceetum, Luzulo luzuloidis-Fagetum, Abieti-Picetum (montanum), Dentario glandulosae-Fagetum*), and under alpine grasslands (*Nardetum strictae*). These soils are acidic or very acidic. The average value of soil pH in the Skrzyczne (1252 m. a.s.l.)–Szczyrk (640 m. a.s.l.) section is 3.8 (H_2_O) and 2.9 (KCl) (plots from 1 to 12, Table 1). Similar values (3.9—H_2_O, 2.9—KCl) were noted on the Klimczok slope under beech and spruce forests (plots: from 13 to 25, Table 1).

The thickness of the soil horizons varies within the study sites due to the degree of slope and the presence of parent rocks on the land surface, as well as conditions that favour the decomposition of plant precipitation (Table 1). Organic carbon content shows variation in organic (O, Olfh) and mineral (A) horizons in both transects (Table 1). All samples ranged in organic horizon from 12.8% (Plot 16—Klimczok-Szczyrk) to 43.1% (Plot 11—Skrzyczne-Szczyrk). The humus horizons are characterized by a very high organic carbon content, with an average value of 12.4%. This is related to the large amount of decomposed organic matter within the mineral mass that forms the humus horizons. Its presence in this horizon is also indicated by the results of the loss of ignition (Table 1).

### Spatial distribution of potentially toxic elements in the studied soils

The occurrence and distribution of potentially toxic elements differ at the sites studied (Table 2). The highest Fe content was found in Klimczok-Szczyrk (plots 23—(21.47 ± 0.54 g kg^−1^ and plots 22—20.2 ± 0.43 g kg^−1^), and the lowest at plot number 1 (2.2 ± 0.3 g kg^−1^, Skrzyczne-Szczyrk). Among potentially toxic elements, lead (Pb) was characterized by high values at most sites, with the highest values recorded in plots: 20 (384.68 ± 0.948 mg kg^−1^, *Prunello-Plantaginetum*), 22 (309.783 ± 0.772 mg kg^−1^, *Dentario glandulosae-Fagetum*), and 19 (302.49 ± 0.466 mg kg^−1^, *Abieti-Picetum (montanum)* in Klimczok-Szczyrk, while the lowest values were recorded in grassland communities (Plot 1, 10; Table 2). Moreover, Zn had varying values in the samples. Its highest content was recorded at three sites (18: 266.46 ± 0.479 mg kg^−1^, 20: 173.663 ± 0.491 mg kg^−1^, and 23: 170.877 ± 1.48 mg kg^−1^—in Klimczok-Szczyrk transect) in the ectohumus horizon (organic layer). In contrast, the lowest values were recorded in the mineral horizon within the *Luzulo luzuloidis-Fagetum* (14: 53.17 ± 0.628 mg kg^−1^) and *Abieti-Picetum (montanum)* (15: 37.12 ± 0.521 mg kg^−1^) communities.

**Table 2:** The concentration of potentially toxic elements in the topsoil along the altitudinal transect.

Sections	Plot no.	Soil horizon	Fe	Pb	Cd	Zn	Cu	Ni	Cr	As	Hg
			g kg^−1^	mg kg^−1^							μg kg^−1^
Skrzyczne–Szczyrk	1*	A	2.2 ± 0.3	47.7 ± 0.42	0.557 ± 0.048	67.743 ± 0.356	5.257 ± 0.057	1.633 ± 0.054	5.93 ± 0.057	5.297 ± 0.037	0.127 ± 0.012
	2	Olfh	4.57 ± 0.21	194.617 ± 0.351	1.153 ± 0.042	113.443 ± 0.572	15.57 ± 0.316	4.683 ± 0.078	8.423 ± 0.176	6.423 ± 0.193	0.273 ± 0.012
	3	A	13.7 ± 0.37	139.217 ± 0.789	0.497 ± 0.017	53.933 ± 0.062	15.063 ± 0.151	5.273 ± 0.021	15.99 ± 0.073	29.24 ± 0.2	0.303 ± 0.017
	4	A	9.7 ± 0.29	123.393 ± 0.444	0.327 ± 0.012	60.417 ± 0.429	15.327 ± 0.288	4.963 ± 0.104	13.987 ± 0.107	21.197 ± 0.282	0.257 ± 0.025
	5	A	17.87 ± 0.74	163.907 ± 0.841	0.47 ± 0.029	71.387 ± 0.453	14.073 ± 0.221	9.187 ± 0.164	20.657 ± 0.43	23.72 ± 0.283	0.307 ± 0.021
	6	A	18.3 ± 0.33	194.053 ± 1.255	0.537 ± 0.021	64.487 ± 0.478	18.59 ± 0.323	7.17 ± 0.277	17.723 ± 0.343	26.38 ± 0.441	0.233 ± 0.017
	7	Olfh	5.9 ± 0.08	94.623 ± 0.451	1.833 ± 0.045	125.633 ± 0.384	19.33 ± 0.33	6.83 ± 0.144	8.93 ± 0.057	7.263 ± 0.142	0.263 ± 0.039
		A	15.5 ± 0.37	122.667 ± 0.493	0.37 ± 0.036	91.347 ± 0.613	11.957 ± 0.098	7.61 ± 0.127	16.823 ± 0.199	21.71 ± 0.314	0.203 ± 0.012
	8	A	14.63 ± 0.33	147.92 ± 0.281	0.41 ± 0.024	75.727 ± 0.355	17.6 ± 0.316	7.623 ± 0.056	17.84 ± 0.432	21.683 ± 0.347	0.247 ± 0.012
	9	A	15.13 ± 0.26	186.463 ± 0.843	0.627 ± 0.029	101.433 ± 0.581	18.02 ± 0.078	8.017 ± 0.069	17.323 ± 0.526	23.85 ± 0.268	0.46 ± 0.022
	10	A	15.47 ± 0.4	46.53 ± 0.42	0.15 ± 0.016	69.887 ± 0.278	11.697 ± 0.229	14.077 ± 0.102	19.613 ± 0.376	5.93 ± 0.08	0.103 ± 0.012
	11	Ol/Ofh	4.1 ± 0.29	73.49 ± 0.39	1.167 ± 0.039	129.847 ± 0.783	15.193 ± 0.233	7.127 ± 0.179	6.923 ± 0.071	3.103 ± 0.11	0.247 ± 0.025
		A	19.3 ± 0.45	188.557 ± 0.442	0.48 ± 0.033	128.263 ± 0.723	24.627 ± 0.441	13.497 ± 0.275	25.74 ± 0.733	25.647 ± 0.394	0.287 ± 0.033
	12	A	17.43 ± 0.42	47.593 ± 0.437	1.02 ± 0.024	154.6 ± 0.471	10.433 ± 0.429	14.717 ± 0.461	21.013 ± 0.05	7.313 ± 0.237	0.083 ± 0.012
Klimczok—Szczyrk	13	Olfh/A	9.83 ± 0.25	221.443 ± 0.514	0.427 ± 0.026	73.443 ± 0.447	16.273 ± 0.551	4.32 ± 0.099	13.427 ± 0.226	20.753 ± 0.2	0.227 ± 0.012
	14	A	12.93 ± 0.26	138.643 ± 0.606	0.257 ± 0.021	53.17 ± 0.628	11.287 ± 0.51	4.11 ± 0.086	13.58 ± 0.441	30.29 ± 0.38	0.173 ± 0.021
	15	Ol/Ofh	8.93 ± 0.29	154.713 ± 0.511	0.94 ± 0.045	136.52 ± 1.189	19.693 ± 0.419	6.94 ± 0.071	13.593 ± 0.321	9.637 ± 0.097	0.353 ± 0.025
		A	16.2 ± 0.24	70.213 ± 0.225	0.107 ± 0.012	37.12 ± 0.521	6.63 ± 0.312	3.057 ± 0.048	12.1 ± 0.636	30.923 ± 0.287	0.107 ± 0.012
	16	Olfh	17.23 ± 0.37	194.477 ± 0.396	0.357 ± 0.021	69.327 ± 0.57	16.373 ± 0.44	5.18 ± 0.204	15.543 ± 0.425	22.253 ± 0.267	0.293 ± 0.037
	17	Olfh/A	6.53 ± 0.34	73.47 ± 0.45	1.02 ± 0.045	129.953 ± 1.099	15.86 ± 0.343	6.22 ± 0.065	9.927 ± 0.23	3.51 ± 0.297	0.217 ± 0.025
		A	16.93 ± 0.29	155.34 ± 0.503	0.31 ± 0.016	72.563 ± 0.504	15.483 ± 0.227	5.39 ± 0.135	16.693 ± 0.425	31.78 ± 0.251	0.32 ± 0.024
	18	Ol	10.57 ± 0.41	125.85 ± 0.873	1.12 ± 0.024	266.46 ± 0.479	19.367 ± 0.533	9.217 ± 0.095	15.353 ± 0.47	13.5 ± 0.147	0.307 ± 0.017
		A	11.77 ± 0.25	126.273 ± 0.499	0.467 ± 0.021	93.543 ± 0.417	13.773 ± 0.095	5.357 ± 0.099	14.21 ± 0.269	18.563 ± 0.405	0.22 ± 0.008
	19	A	18.07 ± 0.45	302.49 ± 0.466	0.54 ± 0.024	130.367 ± 0.605	29.223 ± 0.313	8.96 ± 0.107	22.877 ± 0.256	28.707 ± 0.435	0.52 ± 0.033
	20	Ol/Ofh	6.5 ± 0.37	114.31 ± 0.581	0.943 ± 0.048	173.663 ± 0.491	15.743 ± 0.466	6.983 ± 0.132	10.863 ± 0.455	4.033 ± 0.062	0.263 ± 0.041
		A	15.8 ± 0.67	384.68 ± 0.948	0.84 ± 0.024	142.487 ± 0.827	34.73 ± 0.608	9.497 ± 0.362	22.42 ± 0.331	15.21 ± 0.573	0.637 ± 0.034
	21	Ofh	13.53 ± 0.21	211.43 ± 0.582	0.453 ± 0.012	100.9 ± 0.255	20.637 ± 0.419	6.64 ± 0.313	17.297 ± 0.204	22.86 ± 0.557	0.357 ± 0.033
	22	Ol/Ofh	20.2 ± 0.43	309.783 ± 0.772	0.367 ± 0.029	106.523 ± 0.55	23.69 ± 0.351	8.02 ± 0.062	20.443 ± 0.585	38.573 ± 0.465	0.467 ± 0.021
	23	Ol/Of	9.2 ± 0.24	61.87 ± 0.156	0.723 ± 0.034	170.877 ± 1.481	16.437 ± 0.372	8.497 ± 0.275	12.33 ± 0.586	7.557 ± 0.114	0.15 ± 0.008
		A	21.47 ± 0.54	128.6 ± 0.371	0.307 ± 0.017	85.253 ± 0.801	15.73 ± 0.207	11.287 ± 0.559	23.153 ± 0.833	23.453 ± 0.361	0.167 ± 0.012
	24	A	17.9 ± 0.33	61.64 ± 0.421	0.46 ± 0.016	85.473 ± 0.542	10.61 ± 0.468	13.613 ± 0.478	19.907 ± 0.172	8.703 ± 0.204	0.123 ± 0.012
	25	A	11.87 ± 0.29	128.703 ± 0.235	0.96 ± 0.037	142.597 ± 0.467	18.587 ± 0.325	8.217 ± 0.095	16.067 ± 0.72	13.743 ± 0.325	0.303 ± 0.021

*Explanation: plot numbers and the vegetation they represent: 1—*Nardetum strictae,* 2,3,13—*Plagiothecio-Piceetum,* 4–6, 9, 14, 20—*Luzulo luzuloidis-Fagetum,* 7, 8, 11, 15, 18, 19, 21—*Abieti-Picetum (montanum),* 10- *Prunello-Plantaginetum,* 12—*Prunello-Plantaginetum* and 16, 17, 22–25—*Dentario glandulosae-Fagetum*.

Values exceeding the permitted threshold (25 mg kg^−1^ in Poland) were recorded at seven sites (3: 29.24 ± 0.2 mg kg^−1^, 6: 26.38 ± 0.441 mg kg^−1^ in Skrzyczne-Szczyrk transect, and 14: 30.29 ± 0.38 mg kg^−1^, 15: 30.923 ± 0.287 mg kg^−1^, 17: 31.78 ± 0.251 mg kg^−1^, 19: 28.707 ± 0.435 mg kg^−1^, 22: 38.573 ± 0.465 mg kg^−1^ in Klimczok-Szczyrk section, Table 2), mainly in the mineral horizon (A).

Cd occurred within the accepted standard range, with levels ranging from 0.107 ± 0.012 mg kg^−1^ (level A) to 1.833 ± 0.045 mg kg^−1^ (horizon O). In contrast to cadmium, higher Cr values are found in the mineral level (A, plot: 11—Skrzyczne-Szczyrk & 23, 19—Klimczok-Szczyrk) than in the organic (O) level, ranging from 5.93 ± 0.057 mg kg^−1^ to 25.74 ± 0.733 mg kg^−1^ throughout the study sites (Table 2).

In the case of Ni, the highest contents were found on the lower parts of the slope at 720 m.a.s.l. (14.077 ± 0.102 mg kg^−1^), 680 m.a.s.l. (13.613 ± 0.478 mg kg^−1^), 640 m.a.s.l. (14.717 ± 0.461 mg kg^−1^) on the sites bordering the human settlement (plots 10, 11, 12). The lowest was found in the highest parts of the study area (plots 1, 14 and 15), where its content ranged from 1.633 ± 0.054 to 4.11 ± 0.086 mg kg^−1^ (Table 2).

Cu also occurs within the acceptable standard for forest areas. It accumulates in the humus horizons and does not show much variation in vertical distribution. Higher Cu values were found in A horizons at sites 20 (34.73 ± 0.608 mg kg^−1^), 19 (29.223 ± 0.313 mg kg^−1^), and 11—Skrzyczne-Szczyrk section (24.627 ± 0.441 mg·kg^−1^), represented by *Abieti-Picetum (montanum*) and *Luzulo luzuloidis-Fagetum*. The lowest values (5.257 ± 0.057 mg kg^−1^) were recorded within *Nardetum strictae* community at site 1 at an elevation of 1252 m.a.s.l.

Mercury is present in small amounts at all the sites studied and shows no patterns in distribution. Its highest value was noted at sites 20 (0.637 ± 0.034 μg kg^−1^) and 19 (0.52 ± 0.033 μg kg^−1^) in Klimczok-Szczyrk section, while the smallest amounts were present at sites 12: 0.083 ± 0.012 μg kg^−1^, 10: 0.103 ± 0.012 μg kg^−1^ in Skrzyczne-Szczyrk transect, and 15: 0.107 ± 0.012 μg kg^−1^ (Table 2).

#### Correlation between potentially toxic elements content and soil organic carbon

On the basis of Spearman’s rank correlation coefficient, there was a statistically significant positive correlation in the chemical composition of the soils with respect to potentially toxic elements content between the Fe–Cr, Fe–As, Fe–Ni, Pb–Hg, Ni–Cr, Zn–Cd, Cu–Hg, Cu–Pb, and As–Pb (Table 3). The other relationships between the elements are shown in Supplementary Table S1.

**Table 3:** Correlation in concentration of potentially toxic elements in the analysed soils.

	Fe	Pb	Cd	Zn	Cu	Ni	Cr	As	Hg	pH	C
Fe	1										
Pb	0.331	1									
Cd	− 0.514	− 0.091	1								
Zn	− 0.218	− 0.014	0.734***	1							
Cu	0.125	0.667***	0.302	0.505**	1						
Ni	0.52**	− 0.076	0.153	0.497**	0.267	1					
Cr	0.879***	0.355*	− 0.32	0.027	0.283	0.738***	1				
As	0.683***	0.557**	− 0.617	− 0.481	0.168	− 0.088	0.482**	1			
Hg	0.102	0.772***	0.217	0.285	0.743***	0.052	0.24	0.349	1		
pH	− 0.146	− 0.564	0.365*	0.404*	− 0.224	0.323	− 0.165	− 0.487	− 0.383	1	
C	− 0.544	0.22	0.753***	0.64***	0.564**	− 0.047	− 0.39	− 0.393	0.503**	0.111	1

***p < 0.001; **p < 0.01, *p < 0.05.

There was also a positive correlation between organic carbon content and cadmium and zinc (C–Cd = 0.753, C–Zn = 0.640, C–Cu = 0.564 and C–Hg = 0.503), soil reaction and the content of the elements studied. Only a low value of correlation was found for Zn (r = 0.404), Ni (r = 0.323) and Cd (r = 0.365), other relationships are shown in Table 3 and Supplementary Table S1.

Based on the correlation performed in the altitude aspect in the section from Szczyrk-Klimczok (Table 4), a positive correlation was found between Fe–Cr, Fe–As, Hg–Pb, Cd–Zn and Cu–Hg. Other correlations were found for Cu–Pb (r = 0.657), Pb–Cr (0.529), Cr–Ni (0.593), Pb–As (0.531) and Zn–Cu (0.598, Supplementary Table S2). The remaining correlations are presented in Table 4 and Supplementary Table S2.

**Table 4:** Correlation in the altitude aspect within soil samples.

		Fe	Pb	Cd	Zn	Cu	Ni	Cr	As	Hg
Sectio n Szczyrk–Klimczok	Fe	1								
	Pb	0.346	1							
	Cd	− 0.624	− 0.172	1						
	Zn	− 0.476	− 0.086	0.895***	1					
	Cu	0.011	0.657**	0.461	0.598**	1				
	Ni	0.251	− 0.065	0.441	0.61**	0.399	1			
	Cr	0.810***	0.529*	− 0.199	− 0.03	0.401	0.593**	1		
	As	0.713**	0.531*	− 0.796	− 0.641	− 0.04	− 0.333	0.443	1	
	Hg	0.123	0.816***	0.294	0.379	0.839***	0.203	0.486*	0.212	1
Section Szczyrk–Skrzyczne	Fe	1								
	Pb	0.297	1							
	Cd	− 0.407	0.055	1						
	Zn	0.051	− 0.09	0.569*	1					
	Cu	0.182	0.679**	0.231	0.178	1				
	Ni	0.771**	− 0.13	− 0.275	0.389	− 0.033	1			
	Cr	0.908***	0.134	− 0.429	0.152	0.095	0.899***	1		
	As	0.666**	0.613*	− 0.275	− 0.327	0.415	0.231	0.503	1	
	Hg	0.035	0.669**	0.141	− 0.051	0.554*	− 0.123	0.007	0.506	1

***p < 0.001; **p < 0.01, *p < 0.05.

In the Szczyrk-Skrzyczne section, single correlations were found between individual elements (Table 4) especially for Fe–Ni, Fe–Cr, Cr–Ni, Fe–As, Cu–Pb, Hg–Pb, Cu–Hg, and Zn–Cd (Table 4; Supplementary Table S3). No relationship was found between altitude and the distribution of potentially toxic elements in the study plots.

#### Similarity of potentially toxic elements content of the study plots

**Figure 3:**
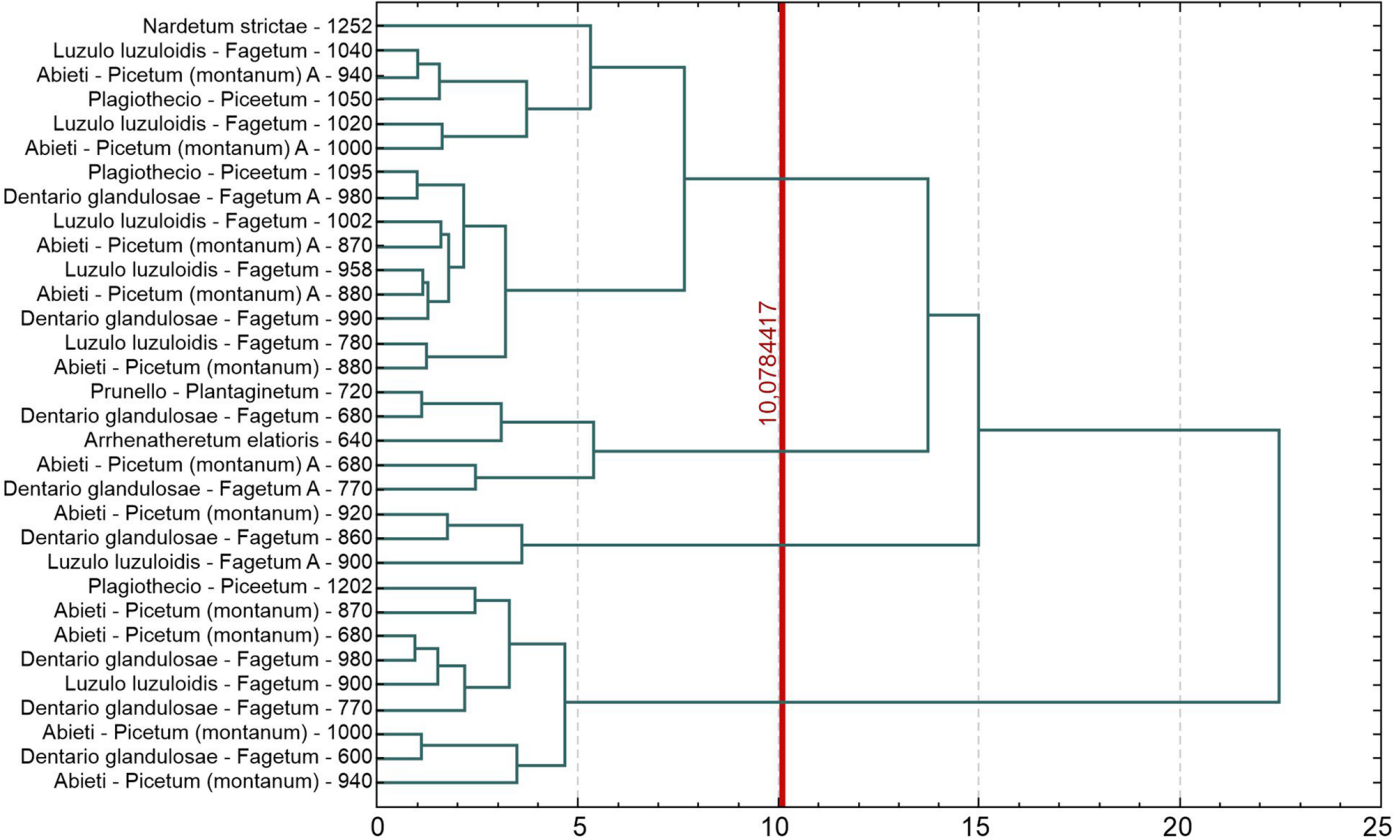
Dendrogram of similarities in metal concentrations under different plant communities.

A similarity dendrogram was created within the samples using Ward’s method^65^, where four clusters were distinguished (Fig. 3).

Cluster I—represents soils occurring under *Abieti-Picetum (montanum), Luzulo luzuloidis-Fagetum, Dentario glandulosae-Fagetum* located at elevations of 920, 900 and 860 m.a.s.l. They were characterized by very high average contents of Pb (32.32), Cu (29.21), As (27.50), and Hg (0.54) compared to the other clusters. Lead and arsenic are significant for this cluster, with contents exceeding the average values for the other clusters many times over. This is the zone where human settlements occur.

Cluster II—includes nine areas (soils) under spruce boreal (*Plagiothecio-Piceetum Abieti-Picetum (montanum*) and beech (*Dentario glandulosae-Fagetum, Luzulo luzuloidis-Fagetum*) communities, which ranged in altitude from 600 to 1202 m.a.s.l. They were characterized by the lowest Fe (0.76), Cr (11.38) and low Hg (0.26) contents among all clusters. This cluster had the highest values of Zn (154.33) and Cd (1.10).

Cluster III—consisted of soils occurring under *Prunello-Plantaginetum, Abieti-Picetum (montanum), Arrhenatheretum elatioris, Dentario glandulosae-Fagetum, Dentario glandulosae-Fagetum* (Fig. 3). These soils were characterized by very low contents of lead (average Pb—94.58) and mercury (Hg—0.15). Low copper content (Cu—14.62) was also observed.

Cluster IV—contained fifteen soils under plant communities located at different altitudes (Fig. 3, Table 1), representing alpine grasslands (*Nardetum strictae*), spruce forests (*Plagiothecio-Piceetum*), mixed fir-spruce forests (*Abieti-Picetum (montanum)*) and beech forests (*Luzulo luzuloidis-Fagetum, Dentario glandulosae-Fagetum*). It was characterized by low soil zinc content (Zn—72.4224) but high arsenic content (As—24.2226) compared to the other clusters.

#### The risk of potentially toxic elements contamination

The calculated Igeo index shows that Pb, As and Cd have the greatest impact on soil contamination in all the areas studied (Table 5). It classifies soils from moderately to highly contaminated. It also shows moderate contamination of Hg and absence of pollution/moderate contamination of Zn in all soil samples (Table 5). The Igeo index calculated for Mn, Fe, Ni, Cu and Cr has a negative or close to zero value, which indicates that these elements do not pollute the soils tested.

**Table 5:** The calculated geo-accumulation index (Igeo) of potentially toxic elements within examined soils.

AMSL [m]	Plot no.	Pb	Cd	Zn	Mn	Fe	Cu	Ni	Cr	Hg	As
600	25	2.33	2.63	0.87	− 3.36	− 1.96	− 0.21	− 1.77	− 1.71	1.82	2.20
640	12	0.91	2.69	0.99	− 0.73	− 1.42	− 1.06	− 0.93	− 1.32	− 0.07	1.30
680	24	1.27	1.59	0.13	− 1.72	− 1.37	− 1.00	− 1.03	− 1.41	0.48	1.55
680	11	1.53	2.91	0.73	− 0.90	− 3.46	− 0.49	− 1.95	− 2.93	1.52	0.05
720	10	0.87	− 0.03	− 0.16	− 2.00	− 1.56	− 0.89	− 0.97	− 1.43	0.21	0.98
770	23	1.28	2.21	1.12	− 2.58	− 2.33	− 0.38	− 1.70	− 2.08	0.87	1.36
780	9	2.87	2.04	0.37	− 3.09	− 1.63	− 0.25	− 1.78	− 1.62	2.41	2.99
860	22	3.60	1.27	0.45	− 2.18	− 1.21	0.14	− 1.78	− 1.38	2.49	3.68
870	7	1.90	3.58	0.69	− 0.49	− 2.95	− 0.16	− 2.04	− 2.56	1.75	1.26
880	8	2.54	1.42	− 0.04	− 3.20	− 1.66	− 0.29	− 1.88	− 1.54	1.58	2.85
880	21	3.05	1.56	0.37	− 2.97	− 1.78	− 0.07	− 2.06	− 1.61	2.05	2.93
900	20	2.16	2.59	1.16	− 0.67	− 2.81	− 0.43	− 2.02	− 2.25	1.67	0.42
920	19	3.57	1.82	0.74	− 2.53	− 1.36	0.44	− 1.65	− 1.19	2.63	3.24
940	18	2.30	2.87	1.77	− 0.49	− 2.14	− 0.17	− 1.60	− 1.79	1.81	2.17
958	6	2.93	1.82	− 0.28	− 3.63	− 1.34	− 0.21	− 1.99	− 1.56	1.48	3.14
980	17	1.52	2.72	0.74	− 1.30	− 2.86	− 0.43	− 2.15	− 2.42	1.29	0.26
990	16	2.93	1.23	− 0.17	− 3.42	− 1.43	− 0.40	− 2.45	− 1.77	1.77	2.91
1000	15	2.60	2.63	0.80	− 2.59	− 2.38	− 0.11	− 2.02	− 1.98	2.10	1.68
1002	5	2.68	1.59	− 0.13	− 3.54	− 1.36	− 0.60	− 1.60	− 1.34	1.87	2.99
1020	14	2.44	0.76	− 0.56	− 3.32	− 1.87	− 0.95	− 2.77	− 1.94	0.99	3.33
1040	4	2.27	1.11	− 0.37	− 4.27	− 2.27	− 0.47	− 2.51	− 1.91	1.54	2.81
1050	13	3.12	1.52	− 0.09	− 2.86	− 2.23	− 0.41	− 2.70	− 1.96	1.46	2.79
1095	3	2.45	1.65	− 0.53	− 4.54	− 1.77	− 0.51	− 2.40	− 1.72	1.89	3.29
1202	2	2.93	2.90	0.54	− 3.74	− 3.33	− 0.48	− 2.57	− 2.64	1.68	1.09
1252	1	0.90	1.92	− 0.20	− 6.30	− 4.40	− 2.03	− 4.12	− 3.15	0.61	0.82

The resulting enrichment factor (Table 6) gives similar results to Igeo. The enrichment of Pb and Cd varies between significant, very high, and extremely high, but these changes are not associated with the altitude at which the samples were taken. The EF obtained for As and Hg indicates significant to very high enrichment in all areas studied. In contrast, EF calculated for Zn indicates moderate to significant enrichment of that element in the soils.

**Table 6:** The enrichment factor obtained for potentially toxic elements within the study sites.

AMSL [m]	Plot no.	Pb	Cd	Zn	Mn	Fe	Cu	Ni	Cr	Hg	As
600	25	19.7	24.2	7.12	0.38	1.00	3.36	1.14	1.19	13.8	17.9
640	12	5.02	17.3	5.31	1.62	1.00	1.29	1.40	1.07	2.55	6.61
680	24	6.26	7.78	2.83	0.79	1.00	1.29	1.27	0.98	3.61	7.59
680	11	31.9	82.9	18.3	5.92	1.00	7.85	2.85	1.45	31.7	11.4
720	10	5.40	2.89	2.64	0.74	1.00	1.59	1.50	1.10	3.41	5.80
770	23	12.2	23.4	11.0	0.84	1.00	3.86	1.55	1.19	9.23	12.9
780	9	22.6	12.7	4.00	0.36	1.00	2.60	0.90	1.01	16.4	24.5
860	22	28.1	5.60	3.16	0.51	1.00	2.56	0.67	0.89	13.0	29.7
870	7	28.7	92.4	12.4	5.51	1.00	6.92	1.88	1.31	25.9	18.5
880	8	18.3	8.45	3.06	0.34	1.00	2.58	0.86	1.08	9.46	22.8
880	21	28.4	10.1	4.44	0.44	1.00	3.28	0.82	1.12	14.2	26.1
900	20	31.4	42.2	15.7	4.40	1.00	5.20	1.74	1.47	22.3	9.36
920	19	30.4	9.04	4.27	0.44	1.00	3.48	0.82	1.12	15.8	24.2
940	18	21.8	32.3	15.07	3.14	1.00	3.93	1.46	1.28	15.5	19.9
958	6	19.3	8.94	2.09	0.20	1.00	2.18	0.64	0.86	7.1	22.3
980	17	20.8	47.8	12.1	2.95	1.00	5.36	1.63	1.35	17.8	8.69
990	16	20.5	6.34	2.39	0.25	1.00	2.04	0.49	0.79	9.2	20.3
1000	15	31.6	32.3	9.09	0.86	1.00	4.82	1.29	1.32	22.3	16.7
1002	5	16.5	7.74	2.35	0.22	1.00	1.70	0.85	1.01	9.44	20.4
1020	14	19.8	6.20	2.48	0.36	1.00	1.89	0.54	0.95	7.25	36.6
1040	4	23.3	10.4	3.73	0.25	1.00	3.49	0.85	1.29	14.1	34.0
1050	13	40.6	13.5	4.41	0.65	1.00	3.53	0.72	1.20	12.9	32.3
1095	3	18.6	10.7	2.35	0.15	1.00	2.39	0.65	1.03	12.6	33.3
1202	2	76.9	75.1	14.6	0.75	1.00	7.24	1.70	1.61	32.3	21.5
1252	1	39.3	79.8	18.3	0.27	1.00	5.15	1.21	2.37	32.1	37.2

As for enrichment in individual plant communities, we can observe that soil enrichment from sites with *Dentario glandulosae-Fagetum* community is mostly slightly lower than in sites with *Abieti—Picetum* (montanum), *Luzulo luzuloidis-Fagetum*, and *Plagiothecio—Piceetum* communities. Furthermore, the level of enrichment in potentially toxic elements is similar for other communities.

Contamination factors show a low level of contamination for Fe, Mn, Cr, and Ni in all areas (Table 7). The CF class of Cu varies between low and moderate factors depending on the sampling site. The contamination factors calculated for Zn show that it causes moderate contamination in all the locations examined. The CF obtained for Hg lies between moderate and very high contamination, with most indicators pointing to considerable contamination. Similarly, Pb, As, and Cd contamination fluctuates between moderate and very high.

**Table 7:** Calculated contamination factors for potentially toxic elements and pollution load index for each study site.

AMSL [m]	Plot no.	Pb	Cd	Zn	Mn	Fe	Cu	Ni	Cr	Hg	As	PLI
600	25	7.57	9.31	2.74	0.15	0.39	1.30	0.44	0.46	5.30	6.90	9.39
640	12	2.81	9.71	2.98	0.91	0.56	0.72	0.78	0.60	1.43	3.70	4.48
680	24	3.63	4.51	1.64	0.46	0.58	0.75	0.74	0.57	2.09	4.40	4.42
680	11	4.33	11.27	2.49	0.80	0.14	1.07	0.39	0.20	4.30	1.55	6.1
720	10	2.74	1.47	1.34	0.37	0.51	0.81	0.76	0.56	1.73	2.95	2.85
770	23	3.64	6.96	3.27	0.25	0.30	1.15	0.46	0.35	2.75	3.85	5.15
780	9	10.99	6.18	1.94	0.18	0.49	1.26	0.44	0.49	7.96	11.90	12.65
860	22	18.21	3.63	2.05	0.33	0.65	1.66	0.44	0.58	8.43	19.20	16.63
870	7	5.58	17.94	2.42	1.07	0.19	1.34	0.37	0.25	5.04	3.60	7.9
880	8	8.70	4.02	1.46	0.16	0.48	1.23	0.41	0.51	4.50	10.85	9.61
880	21	12.42	4.41	1.94	0.19	0.44	1.43	0.36	0.49	6.20	11.40	12.39
900	20	6.71	9.02	3.34	0.94	0.21	1.11	0.37	0.31	4.77	2.00	8.88
920	19	17.79	5.29	2.50	0.26	0.59	2.04	0.48	0.66	9.27	14.15	16.89
940	18	7.39	10.98	5.12	1.07	0.34	1.34	0.49	0.43	5.27	6.75	9.37
958	6	11.43	5.29	1.24	0.12	0.59	1.29	0.38	0.51	4.20	13.20	11.31
980	17	4.31	9.90	2.50	0.61	0.21	1.11	0.34	0.28	3.68	1.80	6.38
990	16	11.43	3.53	1.33	0.14	0.56	1.13	0.27	0.44	5.11	11.30	10.16
1000	15	9.11	9.31	2.62	0.25	0.29	1.39	0.37	0.38	6.41	4.80	9.92
1002	5	9.63	4.51	1.37	0.13	0.58	0.99	0.49	0.59	5.50	11.90	9.69
1020	14	8.15	2.55	1.02	0.15	0.41	0.78	0.22	0.39	2.98	15.05	9.31
1040	4	7.25	3.24	1.16	0.08	0.31	1.08	0.26	0.40	4.38	10.55	8.11
1050	13	13.02	4.31	1.41	0.21	0.32	1.13	0.23	0.39	4.13	10.35	9.88
1095	3	8.19	4.71	1.04	0.06	0.44	1.05	0.28	0.45	5.55	14.65	9.74
1202	2	11.44	11.18	2.18	0.11	0.15	1.08	0.25	0.24	4.80	3.20	10.95
1252	1	2.80	5.69	1.30	0.02	0.07	0.37	0.09	0.17	2.29	2.65	3.44

The potential ecological risk index (PLI) calculated for study sites (Table 8) indicates the existence of pollution in all the areas that were examined. There was no correlation between the CF and PLI values and the height of soil sampling. An analysis of the CF indices for each plant community showed no significant differences. However, a decrease in the value of the PLI index for the *Luzulo luzuloidis-Fagetum* community can be observed along with the altitude of the sample collected.

**Table 8:** Potential ecological risk factor of measured potentially toxic elements and potential ecological risk index (RI) for each study area.

		Pb	Cd	Zn	Mn	Fe	Cu	Ni	Cr	Hg	As	RI
${\text{T}}_{{\text{r}}}^{{\text{i}}}$*		5	30	1	1	1	5	5	2	40	10	
AMSL [m]	Plot no.											
600	25	37.84	279.41	2.74	0.15	0.39	6.48	2.20	0.91	212.14	69.00	611.34
640	12	14.07	291.18	2.98	0.91	0.56	3.60	3.92	1.20	57.14	37.00	412.77
680	24	18.14	135.29	1.64	0.46	0.58	3.75	3.68	1.13	83.57	44.00	292.43
680	11	21.66	338.24	2.49	0.80	0.14	5.34	1.94	0.39	172.14	15.50	558.72
720	10	13.72	44.12	1.34	0.37	0.51	4.04	3.82	1.11	69.29	29.50	168.03
770	23	18.22	208.82	3.27	0.25	0.30	5.75	2.31	0.71	110.00	38.50	388.26
780	9	54.96	185.29	1.94	0.18	0.49	6.30	2.18	0.98	318.57	119.00	689.96
860	22	91.04	108.82	2.05	0.33	0.65	8.29	2.18	1.15	337.14	192.00	743.75
870	7	27.90	538.24	2.42	1.07	0.19	6.72	1.83	0.51	201.43	36.00	816.41
880	8	43.52	120.59	1.46	0.16	0.48	6.14	2.04	1.03	180.00	108.50	464.02
880	21	62.08	132.35	1.94	0.19	0.44	7.16	1.80	0.98	247.86	114.00	568.87
900	20	33.55	270.59	3.34	0.94	0.21	5.56	1.85	0.63	190.71	20.00	527.46
920	19	88.94	158.82	2.50	0.26	0.59	10.20	2.39	1.31	370.71	141.50	777.33
940	18	36.97	329.41	5.12	1.07	0.34	6.68	2.47	0.87	210.71	67.50	661.25
958	6	57.16	158.82	1.24	0.12	0.59	6.47	1.88	1.02	167.86	132.00	527.26
980	17	21.56	297.06	2.50	0.61	0.21	5.55	1.69	0.56	147.14	18.00	494.98
990	16	57.15	105.88	1.33	0.14	0.56	5.67	1.37	0.88	204.29	113.00	490.35
1000	15	45.56	279.41	2.62	0.25	0.29	6.94	1.85	0.76	256.43	48.00	642.19
1002	5	48.16	135.29	1.37	0.13	0.58	4.95	2.47	1.18	220.00	119.00	533.28
1020	14	40.73	76.47	1.02	0.15	0.41	3.88	1.10	0.78	119.29	150.50	394.43
1040	4	36.23	97.06	1.16	0.08	0.31	5.42	1.32	0.80	175.00	105.50	422.97
1050	13	65.11	129.41	1.41	0.21	0.32	5.66	1.16	0.77	165.00	103.50	472.6
1095	3	40.93	141.18	1.04	0.06	0.44	5.27	1.42	0.91	222.14	146.50	559.97
1202	2	57.22	335.29	2.18	0.11	0.15	5.39	1.26	0.48	192.14	32.00	626.27
1252	1	14.01	170.59	1.30	0.02	0.07	1.84	0.43	0.34	91.43	26.50	306.56

${\text{T}}_{{\text{r}}}^{{\text{i}}}$*-the toxic response factor of the metal given by Zhu et al.^64^.

The potential ecological risk for Fe, Mn, Cr, Zn, Ni, and Cu is low (Table 8). The values calculated for Pb vary between low and considerable risk. There is a similar range for arsenic, with most of the soils tested having results belonging to the considerable risk category. The highest risk categories—from considerable to very high—are associated with cadmium and mercury. The potential risk index that assesses the contamination level shows that most of the soils studied can be classified as having a considerable risk of potentially toxic elements impact, and the rest (seven sites) belong to the very high risk category. The elements with the most significant impact on the pollution level were cadmium and mercury. The potential ecological risk of individual potentially toxic elements for the plant communities studied is similar. A difference can be observed for lead, which has a lower potential ecological risk for *Dentario glandulosae-Fagetum* and *Abieti—Picetum* (montanum) communities. It is also possible to notice a decrease in the value of RI for the *Luzulo luzuloidis-Fagetum* community according to the altitude from which the sample was taken.

## Discussion

As a result of many years of human activity and inappropriate forest management, in the mountainous areas of the Polish Carpathians and Sudetes, single-aged spruce trees were introduced where previously composition was dominated by beech. This resulted in the creation of monocultures^29,70,71^. The introduction of spruce into the deciduous and mixed forest habitats of the lower subalpine forest, as a faster-growing species that provided raw material and building blocks for the settlements and industry that were developing at the time, also had an impact on pedological processes^3,72^ and soil chemistry.

### Causes of variation in the basic properties of the topsoils

The transformation of the lower subalpine forests into spruce monocultures resulted in changes in the morphology of soil profiles by forming, among other things, thicker ectohumus horizons (O-Ol-Of). A characteristic feature of the soils studied is the significant thickness of ectohumus (Olfh, Table 1), ranging from 4 to 9 cm. The replacement of the natural deciduous forest community with spruce monoculture has caused and continues to increase the thickness of ectohumus, and also affects other soil properties^73,74^, which can affect the migration and immobilization of potentially toxic elements.

At the study sites, the soil reaction (pH) of the surface soil horizons (organic horizon) under *Plagiothecio-Piceetum* (3.3 in H_2_O, 2.7 in KCl) is often lower than that of soils under *Abieti-Picetum (montanum)* (4.2 in H_2_O, 3.6 in KCl), *Luzulo luzuloidis-Fagetum* (3.7 in H_2_O, 3.0 in KCl), and *Dentario glandulosae-Fagetum* (4.4 in H_2_O, 3.5 in KCl) communities. Such regularity was found in both the Carpathians and the Sudetes^3,74^. The acidic pH in the horizons analysed may be related to the chemical composition of spruce litter (needles, cones, bark, etc.), which contains tannic substances, as well as to mineral composition and weathering processes. The organic (O) and humus (A) horizons in all samples are acidic or very acidic due to the presence of acidic organic matter under beech and spruce forests. Similar soil pH is found in surface levels (pH 3.5–4.5) and also in mineral endohumics (pH 4.5–5.5) in other parts of the Carpathians^74–77^.

The organic carbon content of the sites studied is closely related to the type, rate of delivery of organic matter, and degree of decomposition. Its range varies in all samples tested in organic horizons from 12.8% (plot 16—Klimczok-Szczyrk) to 43.1% (plot 11—Skrzyczne-Szczyrk). In most cases, the surface horizons (mainly organic) consist of poorly decomposed plant material (mainly spruce needles, bark) due to the lack of favourable environmental conditions^78^.

The humus horizons are characterized by a very high organic carbon content, with an average value of 12.4%. This is associated with the large amount of decomposed organic matter within the mineral matter that forms the humus horizons. The loss of the ignition results also indicates its presence at this horizon (Table 1). While the significant amount of organic carbon in the surface soil horizons (O) is closely related to plant litter, its content in the mineral horizons (A) is linked to humified organic matter and to the presence (in the humus horizon) of plant remains covered by mineral material carried from higher territories due to surface runoff^79^. This additional material enriches the humus horizon and is often observed as thin dark grey layers within the layer. Similar observations have been made by other authors studying mountain soils^72,74,76–78^.

### Driving factors of spatial variability of potentially toxic elements

The quantity of potentially toxic elements present in the soil environment should be considered in terms of their presence in the parent rock as a background, determining their possible accumulation in the soil^80^. The elevated potentially toxic elements content may relate to the geological setting. The distribution of these elements may be due to their geochemical mobility and sorption and desorption in surface levels^81^ and anthropogenic sources.

The content of the elements studied in soil samples taken under spruce, beech, and non-forest stands differed in the organic and humus horizons. Often, higher values were found at the organic level (Table 2). This is related to the adsorption capacity of organic matter^81^ and may also be related to the accumulation of potentially toxic elements from the atmosphere by leaves^18,28^.

Among the potentially toxic elements tested, lead (200 mg kg^−1^ value permitted) and arsenic (25 mg kg^−1^) exceeded the acceptable values for this type of ecosystem^82^. In the case of lead, this refers to plots 19 with *Abieti-Picetum (montanum)* (302.4 mg kg^−1^), 20—*Luzulo luzuloidis-Fagetum* (384.6 mg kg^−1^), and 22—*Dentario glandulosae-Fagetum* (309.9 mg kg^−1^) located in the elevation range of 860–920 m.a.s.l. in the Klimczok-Szczyrk transect. The Pb content reported in the literature in soils from Barania Góra, Czantoria and Równica was 155.6–165.6 mg kg^−1^^83^ and shows a similarity to most of the results obtained in this work (Table 2) in contrast to the results recorded by Steindor et al.^18^, according to which Pb values are many times lower (13.3–21.06 mg kg^−1^) from the Silesian and Żywiec Beskids. In Romania (northern part of the Eastern Carpathians), Pb values ranged from 244.9 to 366.2 mg kg^−1^ in the organic and humus layers of acidic brown soils, respectively.

In the Klimczok-Szczyrk, arsenic contents were also found to exceed permissible values, ranging from 28.7 to 38.5 (with a permissible standard of 25 mg kg^−1^)^82^. Increased values of Pb and As were found at the same sites and soil levels (except at site 15). Its presence can be related to the bot natural and anthropogenic origins. Many natural processes, such as volcanic activity, pedogenesis, dust storms and hydrothermal activity, can contribute to the increased arsenic level in soil^84^. Also, arsenic is commonly present in the Earth’s rocks in the form of arsenates, sulfides, sulfosalts, etc., which can releases it into the environment during weathering. It should also be take into account that significant arsenic amounts are released into the environment due to anthropopressure. The sources such as metal and coal mining and smelting, agriculture (pesticide production), and wood preservatives are listed as the most significant anthropogenic sources^84^. The area is developed for winter tourism and related infrastructure, which is one of the sources of soil pollution^7,8,11,37^. The cause of lead and arsenic pollution in these and similar areas is most probably metal-containing dust (metal-bearing dust) from the transport of pollution from industrial and metallurgical plants^28,85^. In the case of the areas studied, taking into account the dominant south-western direction of winds, the content of metals in soils may be influenced by emissions from nearby countries, i.e., the Czech Republic and Slovakia, as well as from the Upper Silesian agglomeration. Mountainous areas are particularly vulnerable to chemical pollution because the high-altitude slopes of mountain ranges are under the influence of more precipitation and take up larger amounts of pollutants leached from the atmosphere^3,86^.

The concentration of Zn and Cu varies at all sites (Table 2). For Zn, higher contents were found in ectohumus mainly in Klimczok-Szczyrk (plots: 15, 17, 18, 20, 23, Table 2) than in mineral horizons (A). In contrast, higher Cu values were found in the humus than in the organic horizon. The concentrations of the elements analysed do not exceed the permissible levels^82^. Similar Zn concentrations are reported for mountain soils from the Bielsko-Biała, Wisła town, and Babia Góra National Park regions, as well as for nature reserves from the Silesian Beskids^19,87^.

The Cu values (range: 5.25–34.7 mg kg^−1^) obtained at the study sites are higher than those reported by Steindor et al.^18^ from the Silesian Beskids, where they range from 5.04 to 6.18 mg kg^−1^. These authors also obtained a different result for Cd (0.98–1.16 mg kg^−1^), in the present study its range was from 0.10 to 1.83 mg kg^−1^. In turn, the results of the analyses show a similarity with those obtained by Kandziora et al.^83^ from Czantoria (1.24 mg kg^−1^) and Barania Góra (1.62 mg kg^−1^), which are areas adjacent to the study area.

Chromium was present in the range of 5.93–25.7 mg kg^−1^, which corresponds to natural conditions^80,82^. Its highest contents were recorded in ectohums. Similar results were obtained in soils from the Magurka Wilkowicka massif region^88^, which is approximately 30 km from the study area. Nickel and mercury are within the range of the permissible standard and did not show a distribution pattern with altitude, as did the other potentially toxic elements analysed at the study sites. The presence of Hg, similar to As, can be linked to human activities such as coal burning and metal ores smelting^89^, as well as incinerators^90^. Elevated Hg content in natural conditions is associated with volcanic activity^89^, and the study area is located outside such activity.

It is worth noticing that the highest concentrations of all potentially toxic elements are found in samples with high organic carbon content. This means that their content is higher in peat and muck horizons and in the ectohumus of forest soils and lower in the surface organic-mineral horizons of turf soils^3^. This variation may also be caused by different densities of organic-mineral matter (Olfh) and peat matter (O/A). This is also confirmed by Spearman’s correlation analysis for Cd (r = 0.753), Zn (0.640), Cu (0.564), Hg (0.503), and Pb (0.220) (Supplementary Table S1).

Analysis of the results showed that the concentration of the potentially toxic elements studied (Pb, Cd, Zn, Cr, Cu, Ni, As) in the Silesian Beskids (in the area of Szczyrk town) was higher (often many times) (Table 2) than the content reported for spruce stands affected by extinction in the Ukrainian Carpathians (Cd—0.14, Cu—2.8, Pb—12.3, Zn—11.0, Ni—1.1, Cr—1.2, Pb—12.3, As—0.0 mg kg^−1^)^34^. The level of potentially toxic elements in soils in other mountain areas in Europe is presented in Table 9. These results show similarity in the case of Pb, Cd, Zn, cu, Ni and Cr. Only a high concentration of As was noted in the Belianske Tatry Mountains (4.2–120.7 mg kg^−1^), while in the Silesian Beskids, the range was 5.23–38.6 mg kg^−1^.

**Table 9:** Total content of potential toxic elements in soils of mountain areas in Europe given in literature.

Location	Author	Fe	Pb	Cd	Zn	Cu	Ni	Cr	As	Hg	Range of concentration
The Western Carpathians	Shparyk and Parpan^34^	2923	12.3	0.14	11.0	2.8	1.1	1.2	0.0	–	Average μg kg^−1^
The Silesian Beskids	Steindor et al.^18^	–	13.3–19.1	0.98–1.16	64.2–134.3	4.5–6.2	–	–	–	–	Range μg kg^−1^
The Silesian Beskids	Kandziora et al.^83^	–	118.6–165.6	0.96–1.62	11.5–37.4	6.6–9.4	–	–	–	–	Range μg kg^−1^
The Silesian Beskids	Ciepał et al.^19^	–	–	–	95.5–170.4	–	–	–	–	–	Range μg·kg^−1^
The Żywiec Beskids	Steindor et al.^18^	–	13.6–21.1	1.10–1.19	176.3–196.0	1.4–1.7	–	–	–	–	Range μg·kg^−1^
Mt. Babia Góra	Ciepał et al.^91^	–	–	–	105.0–215.0	–	–	–	–	–	Range μg·kg^−1^
Mt. Pilsko	Ciepał et al.^91^	–	–	–	185.0–325.0	–	–	–	–	–	Range μg·kg^−1^
The Gorce Mountains	Miśkowiec^92^	–	31.9–57.5	0.22–0.79	56.4–99.9	–	–	–	–	–	Range μg·kg^−1^
The Sądecki Beskids	Dorocki and Korzeniowska^93^	–	17.8–66.6	0.26–0.65	43.2–117.9	8.1–35.8	7.1–44.2	12.5–36.8	–	–	Range μg·kg^−1^
The Sądecki Beskids	Kicińska^94^	–	45.0	1.3	176.0	-	17.0	31.0	5.0	–	Average μg·kg^−1^
the Hăşmaş Mountains (Romania)	Zsigmond and Urak^95^	–	21.9–63.6	0.25–1.93	69.2–242.8	5.6–24.3	–	–	–	–	Range μg·kg^−1^
The Bieszczady Mountains	Kandziora et al.^96^	–	11.8–66.7	0.13–1.75	13.7	2.0–11.4	–	–	–	–	Range μg·kg^−1^
The Tatra Mountains	Kuc^97^	–	–	1.12–2.83	15.9–150.5	–	–	–	–	–	Range μg·kg^−1^
Mt Kasprowy Wierch	Korzeniowska and Krąż^98^	2–4.2%	53.5–117.8	0.3–1.3	61.1–122.5	3.1–12.9	4.6–15.6	25.3–49.2	–	–	Range μg·kg^−1^
Morskie Oko	Korzeniowska and Krąż^98^	3.3–4.9%	114.6–161.1	0.5–1.3	52.2–125.4	9.2–14.8	2.7–5.8	27.5–32.6	–	–	Range μg·kg^−1^
The Belianske Tatry Mountians	Barancokova et al.^99^	–	45.3–199.0	–	–	–	–	–	4.2–120.7	–	Range μg·kg^−1^
the Jizera Mountains	Kváčová et al.^100^	–	163.0	0.82	14.6	15.2	–	–	–	–	Average μg·kg^−1^
European mountain beech forests soils	Štrbac et al.^101^	12–49	1.4–91.8	0.99–6.03	32.5–252.0	11.3–39.8	4.8–56.3	4.2–83.4	1.0–22.9	0.2–5.1	μg·kg^−1^

### Correlation between metals and altitude

The Spearman’s rank correlation coefficient showed the presence a statistically significant positive correlation between Fe–Cr, Fe–As, Fe–Ni, Pb–Hg, Ni–Cr, Zn–Cd, Cu–Hg and Cu–Pb (Supplementary Table S1). These correlations often come from a single site and altitude under similar plant communities. On the other hand, regular correlations were not found between potentially toxic elements content and altitude above sea level. This is also confirmed by the analysis of environmental indicators such as geo-accumulation index (Igeo), enrichment factor (EF) and contamination factors (CF). A study conducted in the Tatra Mountains shows that there is a tendency for potentially toxic elements in soil to decrease with increasing altitude^98^.

Considering the elevation from which the samples were taken, the Igeo values do not show evident decreases or increases with changes in the altitude. If we consider the breakdown of individual vegetation community in relation to altitude, there is still no clear relationship between pollution and elevation. There is also no visible difference in the Igeo values for each community. There were no particularly high differences in trace elements content in soils independent of the vegetation type.

The low EF for Mn, Ni, Cu, and Cr classifies its content as “deficiency to minimal enrichment.” Only in two soil samples significant Cu enrichment was noted. As in the case of the Igeo index, no relationship is observed between potentially toxic elements enrichment of soils and their altitude of occurrence. The values of the environmental indices examined (Igeo, EF and CF) are higher than those of other mountain areas^98,102^ in terms of Cd, Cr, Cu, Ni, Pb and Zn content.

The potential ecological risk index (PLI) calculated for the study sites (Table 8) indicates the presence of contaminants in all study areas. The highest risk categories—from considerable to very high—are associated with cadmium and mercury. The potential risk index (RI), which assesses the contamination level, shows that most of the soils studied can be classified as having a considerable risk of potentially toxic elements impact, and the rest (seven sites) belong to the very high risk category. The level of geo-accumulation index (Igeo) and enrichment factor (EF) in other mountain areas in Europe are presented in Table 10.

**Table 10:** The level of geo-accumulation index (Igeo) and enrichment factor (EF) in soils of mountain areas in Europe given in literature.

Location	Author(s)	Igeo							
		Pb	Cd	Zn	Cu	Ni	Cr	Hg	As
Mountain beech forests across Europe	Štrbac et al.^103^	– 4.62 to 1.44	2.24 to 4.84	− 0.40 to 2.21	− 0.78 to 1.04	− 2.50 to 1.02	− 2.36 to 0.82	1.75 to 6.40	− 10.04 to 1.12
Tatra Mts (Poland)	Korzeniowska and Krąż^98^	1.6	1.9	0.06	− 1.5	2.4	0.0	–	–
Gorce Mts. (Poland)	Miśkowiec^92^	− 1.0 to 2.0	− 3.0 to 2.4	− 1.0 to 1.4	–	–	–	–	–
Mt. Babia Góra (Poland)	Łyszczarz et al.^104^	− 1.59	0.20	− 1.59	− 3.47	–	− 5.68	–	–
Central Caucasus region	Kushnov et al.^105^	− 0.30	− 6.97	0.84	0.14	− 1.25	–	–	–

Location	Author(s)	EF							
		Pb	Cd	Zn	Cu	Ni	Cr	Hg	As
Mountain beech forests across Europe	Štrbac et al.^103^	0.31–20.30	212.21–1291.71	1.14–6.95	4.36–15.41	1.33–15.16	0.59–5.28	201.57–5070.19	0.01–32.69
Tatra Mts (Poland)	Korzeniowska and Krąż^98^	3.9	4.1	1.2	0.4	0.3	1.7	–	–
Gorce Mts. (Poland)	Miśkowiec^92^	3.8–31	5.5–239	0.7–3.9	–	–	–	–	–
Central Caucasus region	Kushnov et al.^105^	1.21	0.01	2.69	1.64	0.62	–	–	–

The analysis of the Igeo index indicates differences in its values in various regions of Europe (Table 10). In beech forests growing in mountainous areas of Europe, the value of this indicator for Pb ranges − 4.6 to 1.44, while in the study area, the value is at the level of 0.87–3.6. The study area is similar to the the Tatra Mountains (1.6) and is characterized by higher values than the Gorce Mountains (− 1 to 2.0) and Babia Góra (− 1.59). The results for Cd, Zn and Cu show values similar to those in European forests, while they are lower for Cr, Ni and Hg (Table 10). In turn, the EF indicators for Cr and As are similar to mountain beech forests throughout Europe; they are lower for Pb, Cd, Ni and Hg, and higher for Zn (Table 10).

## Conclusions

Centuries of human activity have led to changes in the structure of forest communities in the Silesian Beskids, with the beech and fir-beech forests of the time being replaced by artificially introduced common spruce. In recent periods, these ecosystems have been degraded, one of the causes of which is indirectly attributed in part to potentially toxic elements. In the study area, Dystric Cambisol soils develop under both conifers (*Plagiothecio-Piceetum*, *Abieti-Picetum*) and deciduous trees (*Luzulo luzuloidis-Fagetum*, *Dentario glandulosae-Fagetum*). These are acidic or very acidic soils. The thickness of the organic and humic horizons, which affect the accumulation of potentially toxic elements, varies within the study sites, which results from the degree of terrain inclination and the presence of parent rock on the surface.

The analyze of the spatial distribution of potentially toxic elements and the risk of potentially toxic elements contamination based on selected indicators showed that:

- The concentrations of Pb and As were higher than the maximum permissible limits set for a larger number of countries. The remaining elements were within the permissible range. The high Pb and As concentrations in the ectohumus of the soils in the Silesian Beskids were most likely caused by the long-distance transport of anthropogenic emissions.
- The highest concentrations of most of the potentially toxic elements occur in ectohumus, while their low concentrations occur in the surface organic-mineral and mineral horizons. Such a differentiation may result from different density of mineral-organic matter.
- No correlation was found between altitude and the content of potentially toxic elements in soils in the Skrzyczne and Klimczok transects.
- The level of the risk of potentially toxic elements contamination depends on the indicator applied:
- the geo-accumulation index (Igeo) shows that Pb, As and Cd have the highest impact on soil contamination,
- the enrichment factor (EF) show that for Pb and Cd the contamination risk varies between significant, very high, and extremely high, whereas for As and Hg from significant to very high,
- the potential ecological risk index (PLI) indicate the existence of pollution in all the sites studied.

### Supplementary Information

Supplementary Tables.

## Data Availability

The datasets used and/or analysed during the current study are available from the corresponding author upon reasonable request.
