# Supplementary material for: The spatial distribution of potentially toxic elements in the mountain forest topsoils (the Silesian Beskids, southern Poland)
Source: Sci Rep. 2024 Jan 3;14:338. doi: 10.1038/s41598-023-50817-7 (PMC10764751; doi:10.1038/s41598-023-50817-7)
Supplement: Supplementary file 1 — Supplementary Tables. [file 41598_2023_50817_MOESM1_ESM.pdf]

The spatial distribution of potentially toxic elements in the mountain forest topsoils (the Silesian Beskids, southern Poland)

Oimahmad Rahmonov[1], Michał Sobala*[1], Dorota Środek[1], Dominik Karkosz, Sławomir Pytel[1] and Małgorzata Rahmonov[1]

[1]Institute of Earth Sciences, Faculty of Natural Sciences, University of Silesia, Będzińska 60, 41-200 Sosnowiec, Poland

*michal.sobala@us.edu.pl

Supplementary Table S1. Correlation in concentration of heavy metals between Szczyrk and Klimczok section

		Fe	Pb	Cd	Zn	Cu	Ni	Cr	As	Hg	PH	C
Fe	r	1.000	.331	-.514	-.218	.125	.520	.879	.683	.102	-.146	-.544
	p	.	.065	.003	.230	.495	.002	.000	.000	.577	.425	.001
Pb	r	.331	1.000	-.091	-.014	.667	-.076	.355	.557	.772	-.564	.220
	p	.065	.	.620	.940	.000	.678	.046	.001	.000	.001	.227
Cd	r	-.514	-.091	1.000	.734	.302	.153	-.320	-.617	.217	.365	.753
	p	.003	.620	.	.000	.092	.403	.074	.000	.232	.040	.000
Zn	r	-.218	-.014	.734	1.000	.505	.497	.027	-.481	.285	.404	.640
	p	.230	.940	.000	.	.003	.004	.881	.005	.114	.022	.000
Cu	r	.125	.667	.302	.505	1.000	.267	.283	.168	.743	-.224	.564
	p	.495	.000	.092	.003	.	.140	.116	.359	.000	.219	.001
Ni	r	.520	-.076	.153	.497	.267	1.000	.738	-.088	.052	.323	-.047
	p	.002	.678	.403	.004	.140	.	.000	.632	.776	.071	.799
Cr	r	.879	.355	-.320	.027	.283	.738	1.000	.482	.240	-.165	-.390
	p	.000	.046	.074	.881	.116	.000	.	.005	.186	.368	.027
As	r	.683	.557	-.617	-.481	.168	-.088	.482	1.000	.349	-.487	-.393
	p	.000	.001	.000	.005	.359	.632	.005	.	.051	.005	.026
Hg	r	.102	.772	.217	.285	.743	.052	.240	.349	1.000	-.383	.503
	p	.577	.000	.232	.114	.000	.776	.186	.051	.	.030	.003
PH	r	-.146	-.564	.365	.404	-.224	.323	-.165	-.487	-.383	1.000	.111
	p	.425	.001	.040	.022	.219	.071	.368	.005	.030	.	.544
C	r	-.544	.220	.753	.640	.564	-.047	-.390	-.393	.503	.111	1.000
	p	.001	.227	.000	.000	.001	.799	.027	.026	.003	.544	.

Supplementary Table S2. Correlation in the altitude aspect in the section Szczyrk – Klimczok.

		Fe	Pb	Cd	Zn	Cu	Ni	Cr	As	Hg
Fe	r	1.000	.346	-.624	-.476	.011	.251	.810	.713	.123
	p	.	.160	.006	.046	.964	.316	.000	.001	.627
Pb	r	.346	1.000	-.172	-.086	.657	-.065	.529	.531	.816
	p	.160	.	.494	.735	.003	.798	.024	.023	.000
Cd	r	-.624	-.172	1.000	.895	.461	.441	-.199	-.796	.294
	p	.006	.494	.	.000	.054	.067	.428	.000	.236
Zn	r	-.476	-.086	.895	1.000	.598	.610	-.030	-.641	.379
	p	.046	.735	.000	.	.009	.007	.906	.004	.121
Cu	p	.011	.657	.461	.598	1.000	.399	.401	-.040	.839
	r	.964	.003	.054	.009	.	.101	.099	.874	.000
Ni	p	.251	-.065	.441	.610	.399	1.000	.593	-.333	.203
	r	.316	.798	.067	.007	.101	.	.009	.176	.418
Cr	p	.810	.529	-.199	-.030	.401	.593	1.000	.443	.486
	r	.000	.024	.428	.906	.099	.009	.	.066	.041
As	p	.713	.531	-.796	-.641	-.040	-.333	.443	1.000	.212
	r	.001	.023	.000	.004	.874	.176	.066	.	.399
Hg	p	.123	.816	.294	.379	.839	.203	.486	.212	1.000
	r	.627	.000	.236	.121	.000	.418	.041	.399	.

Supplementary Table S3. Correlation in the altitude aspect in the section Szczyrk – Skrzyczne.

		Fe	Pb	Cd	Zn	Cu	Ni	Cr	As	Hg
Fe	r	1.000	.297	-.407	.051	.182	.771	.908	.666	.035
	p	.	.303	.149	.864	.533	.001	.000	.009	.905
Pb	r	.297	1.000	.055	-.090	.679	-.130	.134	.613	.669
	p	.303	.	.852	.759	.008	.659	.648	.020	.009
Cd	r	-.407	.055	1.000	.569	.231	-.275	-.429	-.275	.141
	p	.149	.852	.	.034	.427	.342	.126	.342	.631
Zn	r	.051	-.090	.569	1.000	.178	.389	.152	-.327	-.051
	p	.864	.759	.034	.	.543	.169	.605	.253	.864
Cu	r	.182	.679	.231	.178	1.000	-.033	.095	.415	.554
	p	.533	.008	.427	.543	.	.911	.748	.140	.040
Ni	r	.771	-.130	-.275	.389	-.033	1.000	.899	.231	-.123
	p	.001	.659	.342	.169	.911	.	.000	.427	.675
Cr	r	.908	.134	-.429	.152	.095	.899	1.000	.503	.007
	p	.000	.648	.126	.605	.748	.000	.	.067	.982
As	r	.666	.613	-.275	-.327	.415	.231	.503	1.000	.506
	p	.009	.020	.342	.253	.140	.427	.067	.	.065
Hg	r	.035	.669	.141	-.051	.554	-.123	.007	.506	1.000
	p	.905	.009	.631	.864	.040	.675	.982	.065	.
